# Supplementary material for: Adaptive coping strategies among individuals living with long-term chikungunya disease: a qualitative study in Curaçao
Source: BMJ Open. 2024 Feb 7;14(2):e076352. doi: 10.1136/bmjopen-2023-076352 (PMC10860096; doi:10.1136/bmjopen-2023-076352)
Supplement: Supplementary data [file bmjopen-2023-076352supp002.pdf]

Supplemental File 2. Interview topic guide.

1. Could you introduce yourself, what is your age, the amount of people living with you in your household, and occupation?

Persistent rheumatic symptoms

1. Would you please describe your experience with chikungunya disease, starting from the first symptoms?
2. What are the symptoms that you are still experiencing, since chikungunya infection?
   a) Describe the rheumatic symptoms; joint pain, swelling, stiffness, cramps and/or locking, and body locations.
      i. Are the rheumatic symptoms constant or recurrent (come and go)?
      ii. How long will the recurrent symptoms last?

Living and coping with persistent symptoms

1. In which way does the chikungunya related symptoms and pain interferes with your daily functioning/activities?
2. How do you deal with the physical presentation of chikungunya disease (at present)? What strategies do you have to manage?
   a) How are you managing the physical limitations?
   b) Does spirituality or religion help you? If yes, can you explain how?
3. Are there any moments that the symptoms and/or pain does not affect you? Can you explain how come?
   a) Is there a difference between 1 year, 2 years, and 5 years (use surveys as recollection) post-infection? If yes, what caused the change?
   b) To what extent have you accepted a life with symptoms and/or pain?

Ending

Is there anything else that you would like to say that we may have not discussed or covered already?
