# Supplementary material for: Adaptive coping strategies among individuals living with long-term chikungunya disease: a qualitative study in Curaçao
Source: BMJ Open. 2024 Feb 7;14(2):e076352. doi: 10.1136/bmjopen-2023-076352 (PMC10860096; doi:10.1136/bmjopen-2023-076352)
Supplement: Supplementary data [file bmjopen-2023-076352supp003.pdf]

Supplemental Table 1. Themes, codes, and example quotes according to adaptive coping strategy.

Theme	Codes	Example quotes
**Learning to live with the disease**	Acceptance of symptom	"With time I have learned acceptance and to deal with them [persistent symptoms]." (Pt.2)
	Learning to live with symptoms	"I have learned to live with having it [chikungunya]. I have learned to deal with it". (Pt.3)
	Acceptance through positivity	"I have put in my mind [accepted] that I have chikungunya, when it [chikungunya] has to go [subside] it will go. It's okay with me, as long as it does not make me handicap [fully impaired] or that I cannot do anything". (Pt.9)
	Minimizing emotional/mental impact	"I cannot say that I worry about it [mentally]…because I have it already [persistence of symptoms]. You [talking in third person] need to learn to live with everything that you have, you need to learn how to deal with the situation". (Pt.7)
**Resilience for dealing with pain**	Pain resilience	"I will not let the pain hamper me in anything that I want to do. For example, if tomorrow I want to climb the Mount Christoffel [highest point of Curaçao], I will go with of without pain. […]. I will not let the pain break me". (Pt.16)

	Ignoring pain sensations	“I will just ignore the symptoms, I will pretend that they [symptoms] are not there. I need to do my things, so I will pretend that I am not in pain”. (Pt.17)
	Mind-body connection	“Your mind heals you, your mind kills you. We have to watch out carefully, I wish everybody knew that. Your mind has to liberate from it all, if you feel that you always have the thing [symptoms] in your mind, you will feel it [symptoms] more than you have it”. (Pt.15)
**Maintaining a positive self-image and attitude**	Positive self-image	“I will wake up and say, that today I am better than yesterday. Today I will accomplish more than I did yesterday…It [doing that] fills me with self-worth”. (Pt.15)
	Positive attitude	“I always think that life goes on and in life you need to keep going forward. […]. When the pain is less, go out [socially] to relax yourself”. (Pt.7)
	Social comparison	“I think that you can learn from the mistakes of others. I often see that a problem can become a stress or depression for others. When I see that, I say that I do not want to become that way [stressed or depressed]. […]. They let a small problem take their minds over”. (Pt.19)
**Coping through spirituality**	Finding strength and courage	“I tell God everyday, that I need to be able to go to work, give me strength to get to work and come back home”. (Pt.20)
	Control through prayer and faith	“I have asked God to not wake up with pain in the morning and I woke up without pain”. (Pt.5)

	Guidance medical knowledge and care	“The only thing that I hope is that he [God] gives the doctors medical knowledge to help me”. (Pt.1)

Pt; participant.
